# Variation in the prevalence of cough symptoms 4–5 days after infection with SARS‐CoV‐2 between seasons with different prevalent strains

**DOI:** 10.1002/jgf2.536

**Published:** 2022-03-09

**Authors:** Tetsuya Akaishi, Tadashi Ishii

**Affiliations:** ^1^ Department of Education and Support for Regional Medicine Tohoku University Hospital Sendai Japan

**Keywords:** coronavirus disease 2019 (COVID‐19), cough symptoms, severe acute respiratory syndrome coronavirus 2 (SARS‐CoV‐2), variant

## Abstract

**Background:**

The coronavirus disease 2019 (COVID‐19) pandemic caused by severe acute respiratory syndrome coronavirus 2 (SARS‐CoV‐2) remains a major global health concern in 2022. The association between the rapid spread of the variants, which eliminated the original strain, and clinical manifestations with the variants remains undetermined.

**Methods:**

This was a population‐based longitudinal cohort study. Whole citizens in a city with approximately one million population who had contacted COVID‐19 patients and were tested by nasopharyngeal SARS‐CoV‐2 reverse transcription‐polymerase chain reaction (RT‐PCR) swab test between July 2020 and March 2021 were enrolled. Detailed contact episode and the presence of cough symptoms 4–5 days after contact with patients having COVID‐19 were evaluated.

**Results:**

Among the 359 RT‐PCR test‐positive patients, 88 (24.5%) developed cough symptoms by 4–5 days from the infection. The same rate in RT‐PCR test‐negative cases was 8.6%. The prevalence of cough did not significantly differ by age, sex, and places or closeness of the contact episode. The rate of cough symptoms in RT‐PCR test‐positive patients increased in February–March 2021 with E484K variant predominance compared to that in July–December 2020 with the original strain (32.9% vs 19.4%, *p* = 0.0221), whereas the cough prevalence among RT‐PCR test‐negative population did not increase. Cough symptoms in COVID‐19 patients was associated with strong fatigability, but was independent from fever or dysosmia.

**Conclusions:**

Cough symptoms 4–5 days after infection with SARS‐CoV‐2 was suggested to have increased with E484K variant, compared to the original strain.

## Graphical abstract

Cough prevalence 4–5 days after infection with COVID‐19 was compared between seasons with the original strain and the subsequent variant. The prevalence of cough 4–5 days after infection increased after the predominant original strain in the study location was replaced by the E484K variant. The prevalence of cough in RT‐PCR test‐negative individuals did not change during the study period.

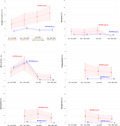

## 1 INTRODUCTION

The coronavirus disease 2019 (COVID‐19) pandemic caused by severe acute respiratory syndrome coronavirus 2 (SARS‐CoV‐2) remained a major global health concern in 2021.^1^ The continuous appearance and spread of new consequential variants has intermittently caused a large‐scale pandemic.^2^, ^3^, ^4^ In parallel with the intermittent appearance of consequential variants, many factors, such as predominant variants, prevalence, and social circumstances, have dynamically changed after the onset of the COVID‐19 pandemic in various countries, including Japan. In 2020, Japan's major prevailing viral strain was the original strain that has been replaced by other strains of E484K or N501Y lines since February 2021. Subsequently, the dominant strains were replaced by the Delta and Omicron variants.^5^ These consequential variants with increased transmissibility spread faster, but the reasons and mechanisms of their rapid spread have not yet been fully elucidated. For example, the symptoms of SARS‐CoV‐2 test‐positive individuals in the early stages of the disease may have varied over time. Therefore, we aimed to investigate the difference in the symptoms of COVID‐19 between the early and later phases of the pandemic to elucidate the symptom characteristics among patients with COVID‐19. This population‐based cohort study focused on the key COVID‐19 symptoms 4–5 days after infection and compared the incidence of each symptom between different phases of the pandemic.

## 2 METHODS

### 2.1 Participants

The participants were the all individuals in Sendai City, Japan, who had contacted COVID‐19 patients and checked for the infection by SARS‐CoV‐2 reverse transcription‐polymerase chain reaction (RT‐PCR) test between July 2020 and March 2021. The population of the city was approximately 1.1 million during the study period. None of the participants were vaccinated against COVID‐19 during the study period. The participants were tested at 4–5 days after their contact episodes. The nasopharyngeal swab sampling was performed in a single mass screening test center, which was founded by the local governments. During the study season, the original strain was predominant before January 2021, whereas E484K variant was confirmed to have comprised of more than 80% of the infection in February–March 2021 according to sampling test for the genome sequence. The RT‐PCR test was performed by detecting the nucleocapsid protein set no. 2 (N2) gene. The primer/probe set developed by the National Institute of Infectious Diseases in Japan (NIID_2019‐nCoV_N_F2, R2, and P2) was used.^6^

### 2.2 Closeness of contact

A history of close contact with COVID‐19 patients was judged by the local government staff in public health centers based on the fulfillment of all of the following four criteria: (1) contact from 2 days before to 14 days after the onset of symptoms or the positive RT‐PCR test results; (2) not wearing masks; (3) <1 m distance; and (4) ≥15 min of contact. These criteria were not changed during the whole study period.

### 2.3 Evaluated outcomes

Information regarding the age, sex, RT‐PCR test results, and place and closeness of the contact episode were collected in advance upon registration before sampling. Body temperature and the presence of COVID‐19‐related key symptoms (cough, dyspnea, body temperature ≥37.5°C, fatigability, dysosmia, and dysgeusia) were self‐reported and recorded at the time of the nasopharyngeal swab test, scheduled 4–5 days after contact with patients having COVID‐19.

### 2.4 Statistical analysis

Comparisons of the prevalence data were performed by chi‐square test or Fisher exact test, according to the number of individuals in each cell. Comparisons of the quantitative data were performed by the Student's *t*‐test or Mann–Whitney *U* test, according to the distribution patterns of the variables. *p* = 0.05 was used for the statistical significance. For each of the key COVID‐19 symptoms, characteristics for estimating SARS‐CoV‐2 RT‐PCR test positivity, such as sensitivity, specificity, positive predictive value, and negative predictive value, were calculated. The Wald method was used to estimate the 95% confidence intervals for the incidence of key COVID‐19 symptoms in RT‐PCR test‐positive and test‐negative individuals.^7^ Statistical analyses were performed with R Statistical Software (version 4.0.5; R Foundation).

### 2.5 Ethics

The institutional review board of Tohoku University approved the present study (IRB approval number: 2021‐1‐705). The need for informed consent was waived to minimize the risk of transmission at the testing center. Consent was secured in an opt‐out manner. Both of these processes were approved by the institutional review board. The whole process of this study was conducted in accordance with the Declaration of Helsinki of 1975, as revised in 2013.

## 3 RESULTS

### 3.1 Participants

A total of 5042 individuals (2712 males and 2330 females), who had recently contacted COVID‐19 patients and underwent RT‐PCR screening test performed by the local government between July 2020 and March 2021 were enrolled in this population‐based longitudinal observational study. Among them, 2639 (52.3%) were with high‐risk contact history and 2403 (47.7%) were with low‐risk contact history. All tested individuals were collected their nasopharyngeal swab samples at a single testing center located in the city. Among the 5,042 tested individuals, 359 (7.1%) with 194 males and 165 females were with SARS‐CoV‐2 RT‐PCR test‐positive results.

### 3.2 Cough symptoms during the whole study periods

In the whole 359 RT‐PCR test‐positive individuals, cough symptoms 4–5 days after infection was seen in 88 (24.5%) of them. The rate of cough symptoms was 18.3% (*n* = 11/60) in the patients aged <18 years, 25.3% (*n* = 65/257) in the patients aged 18–64 years, and 28.6% (*n* = 12/42) in the patients aged ≥65 years. The rates did not significantly differ between the age groups (*p* = 0.4282, chi‐square test). The rate of cough symptoms was 23.2% (*n* = 45/194) among the male patients and 26.1% (*n* = 43/165) among the female patients, which did not differ (*p* = 0.5294). The rate of cough symptoms was 23.0% (*n* = 59/256) among the RT‐PCR test‐positive individuals after a high‐risk contact, and was 28.2% (*n* = 29/103) among the test‐positive individuals after a low‐risk contact. The rate of developing cough symptoms 4–5 days after infection did not significantly differ by the closeness of the preceding contact episode (*p* = 0.3088). The rate of cough symptoms 4–5 days after infection among RT‐PCR test‐positive cases in those who had household infection from other family members was 25.0% (*n* = 30/120), which was not significantly different from the rate among the whole patients after a high‐risk contact or among the patients after a low‐risk contact.

In the 4,683 RT‐PCR test‐negative individuals, cough symptoms 4–5 days after the contact history was seen in 404 (8.6%) of them. The rate of cough symptoms was 11.7% (*n* = 223/1898) in the test‐negative individuals aged <18 years, 6.6% (*n* = 163/2463) in those aged 18–64 years, and 5.6% (*n* = 18/322) in those aged ≥65 years. The rate of cough symptoms was 7.3% (*n* = 174/2,383) among the RT‐PCR test‐negative individuals after a high‐risk contact, and was 10.0% (*n* = 230/2298) among the test‐negative individuals after a low‐risk contact. The rate of cough symptoms 4–5 days after contact among RT‐PCR test‐negative cases was slightly higher among those who had low‐risk contacts than those who had high‐risk contact (*p* = 0.0010, chi‐square test). This could be because of the background that all people who had high‐risk contact underwent nasopharyngeal RT‐PCR swab test, whereas some of the symptom‐free individuals who had low‐risk contacts with patients may have reserved to take the screening test. This could explain the slightly higher cough prevalence in the RT‐PCR test‐positive cases after a low‐risk contact than in those after a high‐risk contact, and may imply that some asymptomatic people after a low‐risk contact with COVID‐19 patients, who were actually with SARS‐CoV‐2 virus, did not undergo RT‐PCR screening tests. The estimated number of such undiagnosed COVID‐19 patients after a low‐risk contact in the locality during the whole study period would be approximately 25–35 patients (during 9 months per one million population), by using the estimated RT‐PCR test‐positive rate of 3.0–4.0% among asymptomatic individuals after a low‐risk contact with patients. This undiagnosed and not quarantined asymptomatic COVID‐19 patients with a low‐risk contact history is estimated to have comprised of 5–10% of the all COVID‐19 patients in the locality.

The RR of RT‐PCR test positivity for being with cough symptoms 4–5 days after a contact with COVID‐19 patients was 1.6 (95% CI: 0.9–2.7) in those aged <18 years, 3.8 (95% CI: 3.0–4.9) in those aged 18–64 years, and 5.1 (95% CI: 2.7–9.8) in those aged ≥65 years. The risk of developing cough symptoms 4–5 days after infection among RT‐PCR test‐positive patients was higher in adults aged ≥18 years than in non‐adults aged <18 years, suggesting that the rate of asymptomatic COVID‐19 patients might be lower in non‐adults aged <18 years than adults aged ≥18 years.

### 3.3 Cough symptoms during each study period

**FIGURE 1:**
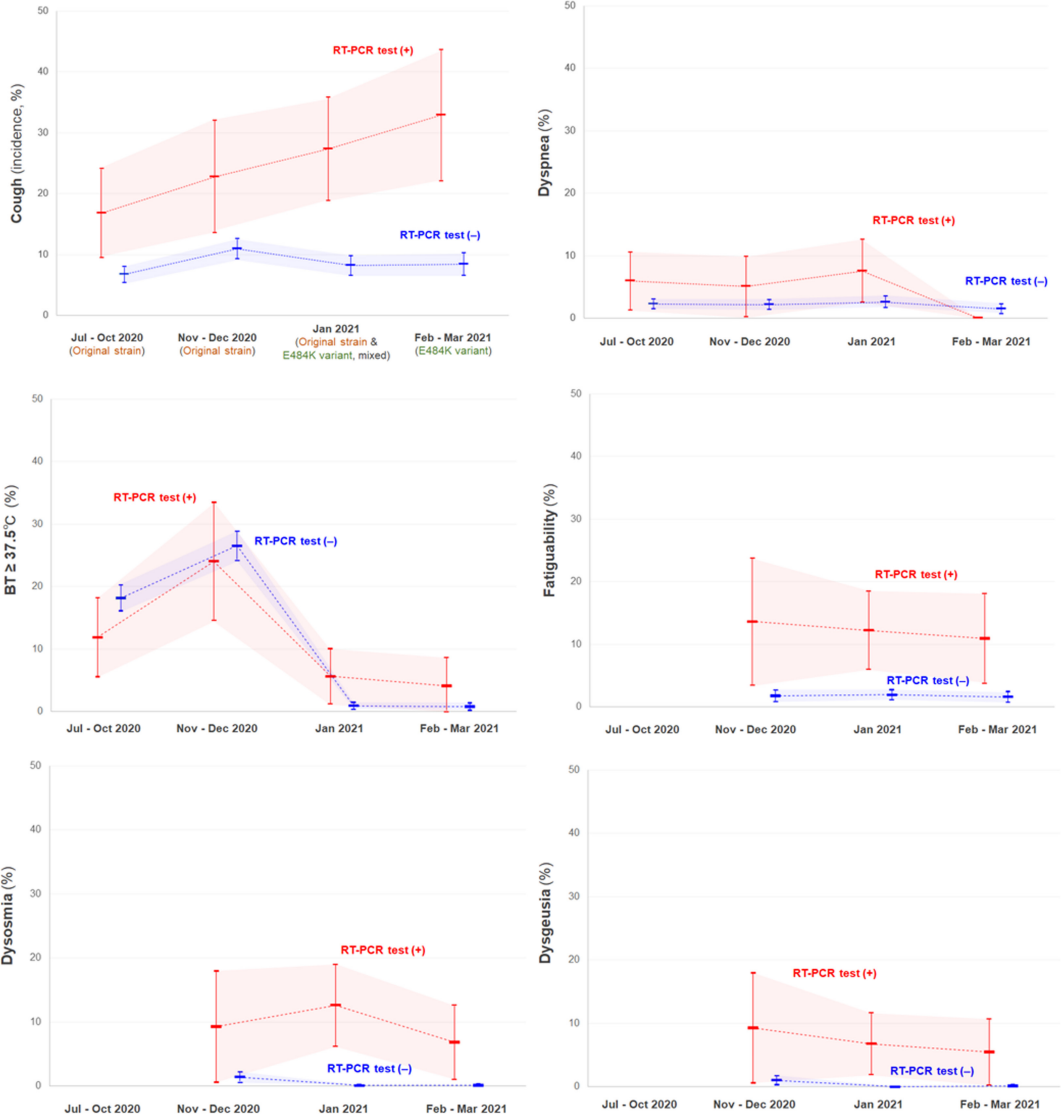
Prevalence of key COVID‐19 symptoms after 4–5 days of contact with patients in RT‐PCR test‐positive and test‐negative individuals. The line graphs suggest that the rate of developing cough symptoms 4–5 days after infection among RT‐PCR test‐positive patients with COVID‐19 might have gradually increased from January 2021, when the prevailing original strain in the locality was gradually replaced by the E484K variant. The incidence of the other evaluated symptoms showed similar patterns of change between the RT‐PCR test‐positive and test‐negative individuals. The filled red or blue areas are the 95% confidence intervals for the prevalence of the evaluated key COVID‐19 symptoms in RT‐PCR test‐positive or test‐negative individuals with a recent contact history with the patients. BT, body temperature; COVID‐19, coronavirus disease 2019; RT‐PCR, reverse transcription‐polymerase chain reaction; SARS‐CoV‐2, severe acute respiratory syndrome coronavirus 2

Next, the prevalence of the aforementioned key COVID‐19 symptoms in RT‐PCR test‐positive and test‐negative individuals in four different seasons was evaluated (Table 1). In all seasons, the prevalence of cough symptoms, fatigability, dysosmia, and dysgeusia was higher in RT‐PCR test‐positive individuals than in test‐negative individuals. The incidence of cough symptoms among RT‐PCR test‐positive individuals gradually increased from January 2021, when the E484K variant gradually replaced the original strain. Meanwhile, other evaluated symptoms did not significantly increase in the seasons after the original strain was replaced by the subsequent variant. The changes in the rate of the evaluated key COVID‐19 symptoms among RT‐PCR test‐positive and ‐negative individuals in 2020 and early 2021 are shown in Figure 1. The rate of developing cough symptoms 4–5 days after infection among RT‐PCR test‐positive COVID‐19 patients was higher in February–March 2021 with the prevailing E484K variant compared to that in July–December 2020 with the original strain (*p* = 0.0221, chi‐square test).

**TABLE 1:** Predictive impacts of each key COVID‐19 symptom for RT‐PCR test positivity

	RT‐PCR (+) (with: without the symptom, *n*)	RT‐PCR (−) (with: without the symptom, *n*)	Sensitivity of each symptom	Specificity of each symptom	PPV	NPV
July–October 2020 (original strain predominant)						
Cough	17: 84	90: 1247	0.168	0.933	0.159	0.937
Dyspnea	6: 95	30: 1307	0.059	0.978	0.167	0.932
BT ≥ 37.5°C	12: 89	243: 1094	0.119	0.818	0.047	0.925
Fatiguability	Not checked	Not checked	Not checked	–	–	–
Dysosmia	Not checked	Not checked	Not checked	–	–	–
November–December 2020 (original strain predominant)						
Cough	18: 61	152: 1235	0.228	0.890	0.106	0.953
Dyspnea	4: 75	30: 1357	0.051	0.978	0.118	0.948
BT ≥ 37.5°C	19: 60	367: 1017	0.241	0.735	0.049	0.944
Fatiguability	6: 38	14: 779	0.136	0.982	0.300	0.953
Dysosmia	4: 39	11: 771	0.093	0.986	0.267	0.952
January 2021 (unknown mixed rate of the original and E484K variant)						
Cough	29: 77	89: 999	0.274	0.918	0.246	0.928
Dyspnea	8: 98	28: 1060	0.075	0.974	0.222	0.915
BT ≥ 37.5°C	6: 100	10: 1076	0.057	0.991	0.375	0.915
Fatiguability	13: 93	21: 1067	0.123	0.981	0.382	0.920
Dysosmia	13: 90	1: 1042	0.126	0.999	0.929	0.920
February–March 2021 (E484K variant predominant)						
Cough	24: 49	73: 796	0.329	0.916	0.247	0.942
Dyspnea	0: 73	13: 856	0.000	0.985	0.000	0.921
BT ≥ 37.5°C	3: 70	7: 863	0.041	0.992	0.300	0.925
Fatiguability	8: 65	14: 855	0.110	0.984	0.364	0.929
Dysosmia	5: 68	1: 826	0.069	0.999	0.833	0.924

Note

In this study, individuals with positive SARS‐CoV‐2 RT‐PCR test results were regarded as having COVID‐19. The presence of each key COVID‐19 symptom, checked at the nasopharyngeal swab test site, was evaluated for its predictive impact in estimating COVID‐19 infection.

Abbreviations: BT, body temperature; NPV, negative predictive value; PPV, positive predictive value; RT‐PCR, reverse transcription‐polymerase chain reaction; SARS‐CoV‐2, severe acute respiratory syndrome coronavirus 2.

Next, the changes in the prevalence of cough symptoms 4–5 days after infection among the RT‐PCR test‐positive cases were evaluated after dividing the patients into non‐adults aged <18 years and others aged ≥18 years. Between July 2020 and December 2020 during the pandemic with the original strain, the rate of cough symptoms 4–5 days after infection among the RT‐PCR test‐positive cases was 21.1% (*n* = 4/19) among non‐adults and was 19.3% (*n* = 31/161) among adults. Between February 2021 and March 2021 during the pandemic with E484K variant, the rate of cough symptoms 4–5 days after infection among the RT‐PCR test‐positive cases was 12.5% (*n* = 2/16) among non‐adults and was 38.6% (*n* = 22/57) among adults. The rate of cough symptoms 4–5 days after infection among COVID‐19 patients did not change between the two seasons among non‐adults (*p* = 0.6657, Fisher exact test), whereas it significantly increased after February 2021 among adult patients (*p* = 0.0034, chi‐square test).

### 3.4 Associations between the cough symptoms and other COVID‐19 core symptoms

Lastly, the association between the cough symptoms and other COVID‐19‐related symptoms among the RT‐PCR test‐positive COVID‐19 patients has been investigated. The demographic and clinical features of the COVID‐19 patients with and without cough symptoms 4–5 days after infection are summarized in Table 2. The development of cough symptoms at 4–5 days after infection among COVID‐19 patients was associated with the increased rate of developing dyspnea and fatigability, but was not associated with an increased rate of developing dysosmia or dysgeusia.

**TABLE 2:** Demographic and clinical features of the COVID‐19 patients with or without cough symptoms

	COVID‐19 patients with cough symptoms	COVID‐19 patients without cough symptoms	*p*‐value
Number, *n*	88	271	–
Male: female, *n*	45: 43	149: 122	0.5294
Age, years^a^	31 (22–50) years	26 (21–47) years	0.1064
Aged <18 years, *n* (%)	11/88 (12.5%)	49/271 (18.1%)	0.2228
High‐risk: low‐risk contact, *n*	59: 29	197: 74	0.3088
BT, °C^a^	36.7 (36.3–37.1) °C	36.6 (36.3–37.0) °C	0.3410
BT ≥ 37.5°C, *n* (%)	28/88 (31.8%)	75/271 (27.7%)	0.4554
Dyspnea, *n* (%)	12/88 (13.6%)	6/271 (2.2%)	0.0001
Fatiguability, *n* (%)	15/64 (23.4%)	12/159 (7.5%)	0.0010
Dysosmia, *n* (%)	7/64 (10.9%)	15/156 (9.7%)	0.8061
Dysgeusia, *n* (%)	5/64 (7.8%)	10/156 (6.5%)	0.7700

Note

The *p*‐values are the results of chi‐square test or Fisher exact test for the frequencies, and the results of Mann–Whitney *U* test for the quantitative variables.

Abbreviations: BT, body temperature; COVID‐19, coronavirus disease 2019.

Median and interquartile range (25–75 percentiles).

## 4 DISCUSSION

Similar to other countries, Japan has also faced intermittent nationwide pandemic of COVID‐19 infection with multiple consequential variants in 2021. Most of the consequential variants had mutations in genes encoding spike protein, supposedly causing an increased transmissibility after a contact with patients. Increased ability of immune escape in variants could be a major factor that facilitated the increased transmissibility of the variants.^8^, ^9^ The results of this study suggested that an increased prevalence of cough symptoms in the early disease stages could be another factor that could have facilitated the increased transmissibility of the variants. By now, it remains controversial whether the incidence of symptoms was changed with variants from the original strain. Meanwhile, some recent studies demonstrated that clinical manifestations of the Omicron variant significantly may differ from those of the previous SARS‐CoV‐2 strains.^10^, ^11^, ^12^ The exact relationship between the development of cough and transmissibility via droplets has not been established yet, as it would be influenced by multiple confounding factors, such as the breathing patterns, particle size, respiratory gas flow in the airway, airway anatomy, and nasopharyngeal viral loads.^13^, ^14^ Meanwhile, some epidemiological studies demonstrated that the transmissibility of asymptomatic cases is significantly smaller than that of the symptomatic cases,^15^, ^16^ although asymptomatic cases also possess high capability of transmission.^17^, ^18^ These facts may imply that an increased incidence of cough in the early disease stages, if any, could have partially contributed to the increased transmissibility of the variants. Further studies are warranted to determine the presence of change in the incidence of COVID‐19 key symptoms in the early stages of the disease caused by viral strains with different S gene mutations.

### 4.1 Limitations

A limitation of this study was that a viral load in sputum from patients with the original strain and with the subsequent variant were not measured. This study only evaluated the prevalence of cough symptoms in the patients, and whether the viral load in the droplets or sputum was increased with the variant or not is unknown. Another limitation of this study was that not all the participants were checked by the whole genome sequencing test. Although the patients in February–March 2021 were mostly comprised of cases with E484K variant, it remains an indirect proof for the types of SARS‐CoV‐2 variants in each patient. Thus, a small fraction of patients with the original strain could be mixed in the patients infected during the fourth period between February 2021 and March 2021.

## 5 CONCLUSIONS

The incidence of cough symptoms 4–5 days after infection with SARS‐CoV‐2 was different between seasons with the predominance of the original strain and the E484K variant. This finding implies that the prevalence of cough symptoms in the early disease stage of the COVID‐19 infection may differ between SARS‐CoV‐2 strains with different S gene mutations.

## CONFLICT OF INTEREST

The authors have stated explicitly that there are no conflicts of interest in connection with this article.
